# Deliberating at a Distance: Evaluating the Feasibility of a Fully Virtual CHAT Exercise Among Low‐Income Communities

**DOI:** 10.1111/hex.70487

**Published:** 2025-11-13

**Authors:** Lydia Perry, Gloria Carmona, Marion Danis, Charo Ledón, Maria Alvarez, Zachary Rowe, Susan Dorr Goold

**Affiliations:** ^1^ University of Michigan Medical School, Center for Bioethics and Social Sciences in Medicine Ann Arbor Michigan United States; ^2^ Department of Learning Health Sciences University of Michigan Medical School Ann Arbor Michigan United States; ^3^ Buenos Vecinos Ann Arbor Michigan United States; ^4^ Clinical Mental Health Counselor at Total Balance LLC Perrysburg Ohio United States; ^5^ Friends of Parkside Detroit Michigan United States; ^6^ Professor of Internal Medicine and Health Management and Policy University of Michigan Ann Arbor Michigan United States

**Keywords:** community‐based participatory research, Covid‐19, decision‐making, deliberation, government assistance, health equity, virtual

## Abstract

**Introduction:**

Alongside software developers, our community–academic partnership adapted an exercise designed for participatory priority setting to engage low‐income community members in online deliberations about health and welfare spending priorities. This study evaluated the feasibility and quality of a fully virtual deliberative exercise using an adapted Choosing All Together (CHAT) tool among low‐income community members.

**Methods:**

Twenty‐one groups including 128 low‐income individuals diverse in age, household size, race/ethnicity and urbanicity used an online version of the CHAT exercise. Sessions included both English‐ and Spanish‐speaking participants. Pre‐ and post‐CHAT surveys assessed participant experiences and views of online public deliberations.

**Results:**

Mean item and scale scores (on a 0–4 Likert scale, where 0 = strongly disagree and 4 = strongly agree) demonstrated that online use of the CHAT exercise was rated as enjoyable (3.4) and easy to do (3.2). Participants rated the information and choices provided as sufficient (3.4), with most participants agreeing that the choices offered in the deliberation were realistic (3.4) and believable (3.4). Participants generally thought they had enough information (2.4). The view of deliberation scale was scored (3.5), showing deliberative quality was rated highly. Participants agreed they gained an understanding of the arguments that opposed their own (3.2), that they had lots of chances to share their views (3.7) and that their views were considered (3.5). Some (2.8) stated that a few people dominated the discussion.

**Discussion:**

Virtual modes of engagement could enable greater group heterogeneity, the participation of less mobile and more remote persons. Online deliberations added increased comfort and convenience for participants. Many participants stated that they expect and prefer online research participation, demonstrating that Covid may have shifted the paradigm. Participants indicated some technology problems, but these did not hinder participation as demonstrated by favourable mean item scores.

**Conclusion:**

Participants found the online CHAT deliberation easy and enjoyable and felt that the choices and information provided to them were sufficient. Despite technological problems, virtual modes of deliberation appear to be well‐received by participants, adding comfort and convenience.

**Patient or Public Contribution:**

DECIDERS, a steering committee of community partners, co‐created the virtual CHAT game and guided its design. Participants shared their stories and provided feedback on the virtual experience, informing both the evaluation and interpretation of the findings.

## 1 Introduction

Health disparities among marginalised groups are exacerbated by the lack of access to social, health and financial resources. Decisions on how to allocate limited resources for health (including to improve social determinants of health) are difficult and can be influenced by political ideologies, research and stakeholders. The people most affected by decisions about social (safety net) programmes typically have less voice in those decisions. However, involving low‐income community members in decisions about priority setting may promote greater justice and equity [1, 2]. Prior research demonstrates that public deliberations may also strengthen knowledge, increase open‐mindedness and improve communication about policy [3, 4].

Traditionally, deliberation sessions have been conducted in person, relying on face‐to‐face interaction to foster trust [5], inclusivity [6, 7] and high‐quality dialogue [8]. However, the Covid‐19 pandemic necessitated a rapid shift to virtual research due to social distancing guidelines. Researchers using participatory methods like deliberations responded by shortening sessions [9], rethinking tools and norms [10, 11], and exploring hybrid and fully virtual formats [12]. Keen et al. argue that adapting qualitative methods with the use of virtual tools should be seen as a longitudinal goal, with some methods, such as focus groups, having an advantage in the virtual environment over traditional in‐person modes [13]. The transition from in‐person participation to virtual formats due to the Covid‐19 pandemic should not be seen as a temporary solution, but rather a new methodological opportunity [14]. Still, the move online has exposed issues such as individuals lacking access to devices, stable internet or the digital literacy required to participate [15, 16]. This is important because those from disadvantaged backgrounds were the most negatively impacted by the Covid‐19 pandemic and are susceptible to ‘digital divide’ or unequal access to digital technology [17, 18].

While published literature indicates that public deliberation can play an important role in providing an avenue for engaging the public in important policy issues, the feasibility and value of conducting deliberation online in virtual meetings have not been well explored or established. Text‐based [19] platforms can reduce real‐time pressure and enhance participation flexibility, but they often lack the richness of conversations in face‐to‐face interaction [20]. Recently, researchers have begun investigating AI‐enhanced qualitative research, including platforms moderated by large language models like ChatGPT‐4 [21, 22]. While these tools can structure large‐scale conversations and enhance efficiency, they introduce concerns about bias, transparency and trust, particularly among populations already wary of AI or marginalisation by digital systems [23, 24].

Less attention has been paid to researching the challenges and benefits of conducting fully virtual deliberations; using audiovisual virtual modes of performing participatory research better mimics the real‐time dialogue of in‐person sessions and allows for nonverbal cues that are essential for respectful deliberation [25]. The cessation of in‐person activities, combined with the widespread availability and skyrocketing use of platforms like Zoom that allow users to communicate with each other using video conferencing, posed an opportunity to examine the value of deliberation in an online setting. This shift allows us to consider the challenges those from marginalised groups face in accessing and participating in these deliberations [26, 27]. While emerging AI‐based and immersive technologies may provide opportunities for education, decision‐making and predictions, these typically aim for individual users, not groups [28, 29, 30]. Furthermore, many of these are not yet optimised for low literacy or low digital literacy users. Our methodology requires only basic software and devices and little preparation. So long as participants find experiences informative and engaging, and principles of deliberative quality are achieved, the method could enable virtual deliberations for communities that often have barriers related to access and digital literacy.

Little research has directly examined how established deliberative tools, such as CHAT (CHoosing All Together), function in fully virtual environments. CHAT engages ordinary persons in informed, inclusive group deliberation about complex and value‐laden allocation decisions. The CHAT exercise consists of three structured rounds: (1) individuals first make personal choices about how to allocate limited resources; (2) the full group, led by a facilitator, then deliberates to reach a shared decision using a single board; and (3) participants return to individual decisions, which may reflect influence or changes in perspective following the group discussion. CHAT was originally developed to engage community members in decisions about prioritisation for healthcare spending. Over the years, CHAT has been modified for use in a variety of contexts, such as crafting affordable health insurance packages [31], prioritising Medicaid spending [32], and healthcare priorities of disabled adult Medi‐Cal beneficiaries [33].

These projects all had the purpose of engaging communities in decisions about spending a broader array of limited resources [34, 35, 36]. Historically, the CHAT exercise has been conducted in‐person with groups of 9–15 participants. Early iterations of the exercise used physical game boards, with later versions converted to a web‐based exercise [37, 38]. Although web‐based CHAT allowed participants to access the game on digital devices, CHAT sessions were still held in‐person and devices were provided. The current version of CHAT we are assessing focuses on government spending decisions after the Covid‐19 pandemic. This project marks the first time CHAT has been adapted into a fully virtual experience. Due to its novelty, we aimed to measure whether virtual sessions were an adequate way of conducting public deliberations while also looking at their usability and accessibility.

This study fills a critical gap by adapting CHAT into a fully virtual experience focused on engaging the public in deliberations about government spending in the post‐pandemic era. Using accessible audiovisual technology, the study emphasises transparency, usability and human‐to‐human interaction, avoiding dependence on AI or immersive environments that may inadvertently exclude vulnerable groups. In doing so, it aims to evaluate whether a fully virtual CHAT can maintain deliberative quality while promoting equity, inclusiveness and meaningful public participation in complex policy decisions.

## 2 Materials and Methods

### 2.1 Adaptation of the Deliberation Tool

**Figure 1:**
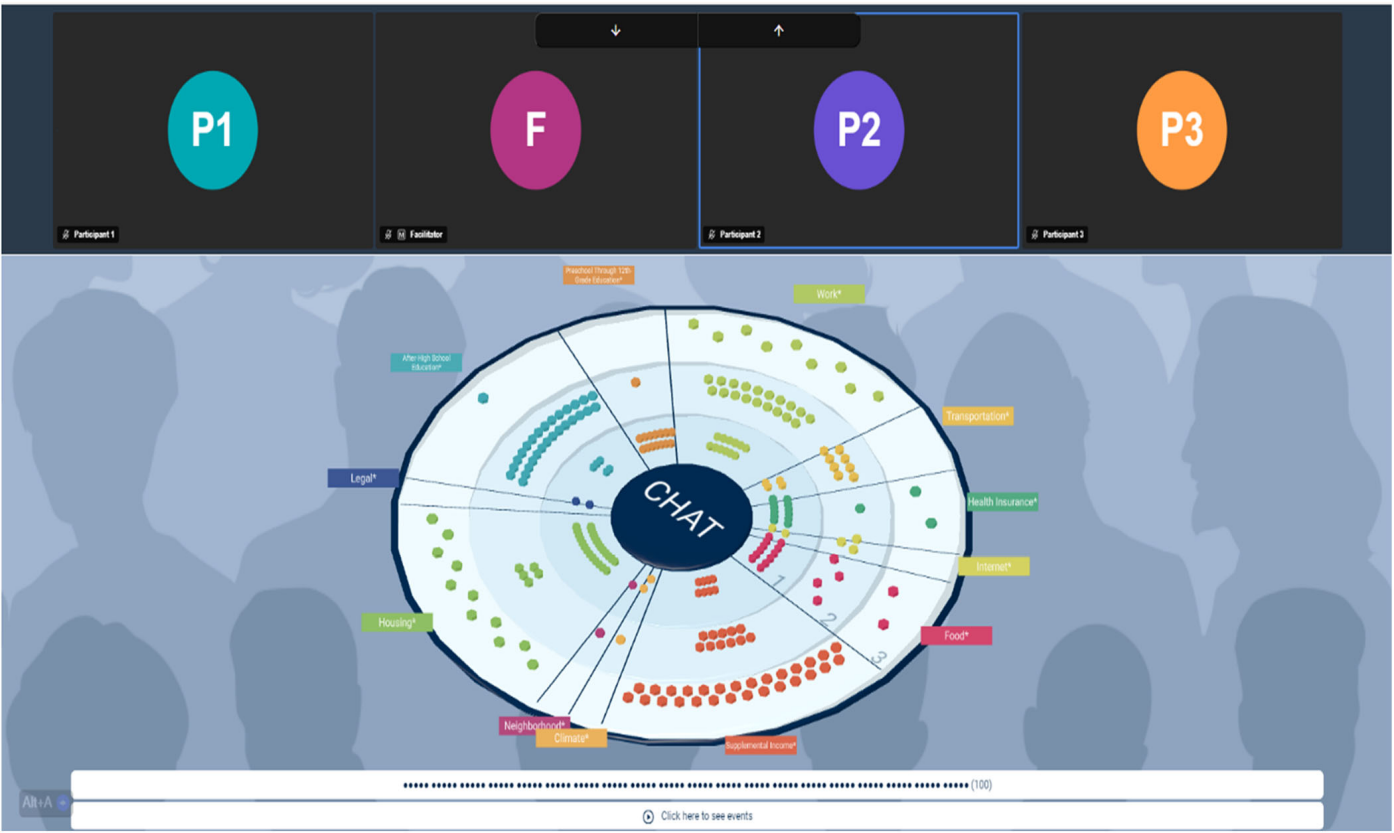
CHAT Board. Screenshot of the CHAT software. The upper portion of the screenshot displays the video feed, which is integrated using Jitsi as a Service or JaaS. The bottom portion displays the CHAT board. The board features 12 categories ranging from 1 to 3 levels of spending. At the bottom of the screen, the marker count indicates that 100 markers are available for spending.

The adaptation of CHAT content into a tool for priority setting involved community partners, actuarial data analyses and online resources. We partnered with software developers at YETi CGI [39] to integrate video conferencing into a webpage with the CHAT exercise (Figure 1). The game board portion of the screen is hosted through CasualOS, a website maintained by the non‐profit Casual Simulation, which is free of charge and licensed by MIT. CasualOS is an integration of multiple pieces of open software to promote easier collaborative work [40]. Video conferencing was embedded into CasualOS using Jitsi as a service, or JaaS [41]. The combination of CasualOS and JaaS allowed the full experience to be loaded in one tab, preventing participants from needing to navigate between multiple tabs (Figure 1). We utilised participatory design with the input of our community partners, the DECIDERS Steering Committee [42], throughout the process of co‐creating the software. Community partners suggested hosting a one‐on‐one meeting with each participant to ensure that everyone came to the CHAT session with the same baseline knowledge of how to use CHAT. Therefore, before participating in the CHAT exercise, participants were asked to complete a 30‐min tutorial session to confirm their devices worked with the software and learn how to navigate the web experience. To minimise bias, the tutorial CHAT exercise contained different content (setting priorities for vacation time) from that used during the deliberation session. All study materials, including surveys and CHAT materials, were translated into Spanish. Translations were reviewed for accuracy and clarity by multiple bilingual native Spanish speakers. This study was deemed exempt from review by the University of Michigan Institutional Review Board.

### 2.2 Recruitment

Our study focused on testing that would evaluate whether the tool would be usable and effective across diverse populations, easy to navigate and compatible with affordable devices that run simpler operating systems. We aimed to recruit even numbers of men and women and to oversample those from racial and ethnic minoritised groups as well as those from rural areas as defined by the 2010 Rural–Urban Commuting Area (RUCA) codes [43]. Recruitment efforts utilised Meta (Facebook and Instagram), the website UMHealthResearch.org, newspapers, and distribution of flyers, emails and text messages by community partners. Volunteers were excluded if they reported a too high household income (household income ≥ 300% of the Michigan poverty level) or did not have access to the internet and a desktop, laptop or Chromebook. Initially, volunteers were excluded if they did not live in Michigan. However, initial enrolment was lower than expected for the Spanish‐speaking sessions, so we broadened eligibility criteria to include those residing in the United States outside of Michigan. Groups of 3–9 participants, 21 groups conducted in English and 5 in Spanish, took part in fully virtual deliberations from October 2022 to August 2023.

### 2.3 Data Collection

Multiple data sources included survey facilitators' observations of the deliberations and open‐ended questions for participants after the exercise. The screening survey included demographic questions to assess eligibility criteria and inquired about experience with videoconferencing and navigating web browsers. The pre‐survey was completed after a 30‐min tutorial session, but before the actual CHAT session. The post‐survey was completed after the CHAT session and included questions about the quality of the deliberations. Participants were also asked about their preferences for in‐person versus online sessions, if the tutorial session was helpful, and what aspects of the exercise could be improved upon. After the CHAT sessions were completed, facilitators prompted participants by asking how they felt about completing the CHAT session online versus face‐to‐face and the conversations were recorded.

The Views of Deliberations scale (Cronbach's *α* in previous projects ~0.80) captures participants' overall assessment of the deliberative process, including aspects such as mutual respect, civility, quality of argumentation, and fairness [44, 45]. Other items adapted from prior studies [46, 47] assessed the adequacy of information, range of choices and whether they would trust the process to inform decisions.

### 2.4 Data Preparation and Analysis

Descriptive results include means and standard deviations for individual survey items and scale scores. Mean scores were reported using Likert scales from 0 to 4 where 0 was ‘Strongly Disagree’ and 4 was ‘Strongly Agree’. We also analysed survey responses to questions about technology improvement suggestions and preferences about virtual sessions and tutorial participation. We aimed to see if perceptions of deliberative quality and evaluation of the exercise were comparable to previous in‐person CHAT evaluations.

To report equality of participation, we used the Herfindahl–Hirschman Index (HHI) [48], normalising the calculation based on group size [49]. The normalised HHI, an economic measure, ranges from 0 to 1, with 1 representing complete monopoly and 0 representing completely equal participation. Recordings were available from 22 of the 26 sessions, with 4 sessions having technical difficulties in recording. The recordings were used to count how many instances each participant spoke during the group deliberation round of CHAT (Round 2) and the wrap‐up questions at the end of the exercise.

Sessions were recorded and transcribed verbatim using Rev.com and a transcriptionist. The Spanish‐speaking sessions were both transcribed and translated using the service Datagain.com and were then checked in full by bilingual facilitators. Participants were asked ‘what did you think about the experience of discussing and making decisions together over the Internet versus face to face?’ towards the end of the session. Thematic analysis of these responses was conducted following Braun and Clarke's six‐phase framework [50]. This process involved an initial reading of all responses to gain familiarity with the data, followed by open coding to identify recurring phrases or concepts. Codes were then grouped into broader categories and then refined into structured themes. This approach allowed for the identification of key issues and perceptions consistently raised by participants, providing deeper insight into their collective experiences.

## 3 Results

A total of 128 participants in 26 groups completed the CHAT exercise. Participants were diverse in age, race/ethnicity, educational background and household characteristics (Table 1). Most identified as female (71.1%), and over half were aged 36 or older (54.7%). The sample included participants from rural areas (10.9%) and a little over a quarter of individuals identified as Hispanic or Latino (28.1%), not all of whom participated in Spanish‐only sessions.

**Table 1:** Participant characteristics (*N* = 128).

	(%)	Mean (SD)
Sex		
Male	26.6	
Female	71.1	
Other	2.3	
Age (Mean, (Range))		38.3 (20–68)
20–35	45.3	
36+	54.7	
Race/Ethnicity*		
White	51.6	
Black or African American	25.8	
Asian	17.2	
Other	9.4	
American Indian or Alaska Native	7.8	
Native Hawaiian or Pacific Islander	0.8	
MENA (Arab, Chaldean, Middle Eastern and/or North African)	2.3	
Hispanic	28.1	
Non‐Hispanic, Non‐MENA white	28.9	
Households with children	42.9	
Households with children aged 0–4	19.5	
Households with children aged 5–12	32.0	
Households with children aged 13–17	16.4	
Educational attainment		
HS/GED or less	7.0	
Some college or a 2‐year degree	30.5	
4‐year degree or more	62.5	
Rural resident (*RUCA 4 or higher)*	10.9	
Marital status (*n* = 127)		
Single	47.2	
Married or partnered	42.5	
Separated, divorced or widowed	11.9	
Covered by health insurance	90.6	
Health insurance type/source (*n* = 116)		
Job/union	26.0	
Insurance purchased by you or someone else	15.7	
Veteran/VA	0.0	
Tricare/CHAMP	0.0	
Medicaid	35.4	
Medicare	13.4	
County	0.0	
Student plan	8.7	
Other (Don't Know, Supplement, Tribal Health Services)	4.7	
Respondent health		
Very good/Excellent health	40.5	
Has one or more chronic health conditions	51.6	
Covid in self/family		
Ever tested positive for Covid	52.8	
Was sick with Covid	13.4	
Someone in the household tested positive for Covid	57.5	
Someone in the household is sick with Covid	23.6	
Lost a family member to Covid	20.5	

* Participants could select more than one race and ethnicity.

Mean item and scale scores describe generally favourable views of the information and choices provided (Table 2).

**Table 2:** Participants' views of deliberations.

	Mean (SD, range)
I found doing this exercise enjoyable	3.4 (0.8, 0.0–4.0)
I found this exercise easy to do	3.2 (1.1, 0.0–4.0)
The information given to us was believable	3.4 (0.9, 0.0–5.0)
The choices offered in the exercise were realistic	3.4 (0.9, 0.0–4.0)
We did not have enough information to make good decisions (‐)	1.7 (1.3, 0.0–4.0)
There were choices I would have liked to have seen but didn't (‐)	2.4 (1.4, 0.0–4.0)
Views of deliberation^a^	
A few people dominated the discussions (‐)	2.7 (1.3, 0.0–4.0)
The way in which the group reached its decision was not fair (‐)	3.5 (1.0, 0.0–4.0)
It was generally possible to trust other people who participated	3.2 (0.9, 0.0–4.0)
The decisions were superficial (‐)	3.0 (1.2, 0.0–4.0)
There was too little time to discuss (‐)	3.1 (1.2, 0.0–4.0)
People in the group argued by referring to what would be best for themselves (‐)	2.8 (1.4, 0.0–4.0)
Our discussion included responding to others' arguments	3.0 (1.1, 0.0–4.0)
I gained an understanding of the arguments that opposed my own	3.3 (0.8, 0.0–4.0)
My views were considered and taken into account	3.5 (0.8, 0.0–4.0)
I had lots of chances to share my views	3.7 (0.7, 0.0–4.0)
The participants in the group argued by referring to what would be best and most fair for all people	2.4 (1.5, 0.0–4.0)
All positions were considered with equal respect	3.6 (0.7, 0.0–4.0)
The arguments of the other participants were useful in forming my own position	3.4 (0.9, 0.0–4.0)
During the exercise, I was treated with respect	3.8 (0.6, 0.0–4.0)

(‐) Denotes reverse‐scored items.

^a^ Mean of 13 items; each 5‐point item can range from 0 to 4. Cronbach's *α* = 0.73.

The majority of participants (67.5%) said that they would not have preferred to complete the exercise in person had Covid‐19 not been an issue. Nearly all respondents (97.6%) found the 30‐min tutorial session to be helpful.

The main suggestions for improvement were video quality (14.1%), seeing/hearing presentations (8.6%), and other (10.9%), which consisted mostly of comments on lagging and Wi‐Fi connection issues (Table 3). A little over half of the participants (55.5%) reported that no aspects of the video call technology could have been improved. Most participants (86.6%) found listening to others to be either somewhat easy or very easy.

**Table 3:** Features of video call technology that could be improved in the virtual deliberations. ‘What aspects of the video call technology could have been improved?’.

Features	No	Yes
Audio quality from the device	92.2% (118)	7.8% (10)
Video quality from the device	85.9% (109)	14.1% (19)
Seeing/hearing presentations	91.4% (117)	8.6% (11)
Seeing/hearing other participants	94.5% (121)	5.5% (7)
Time for discussions	92.2% (118)	7.8% (10)
None of the above	44.5% (57)	55.5% (71)
Other	89.1% (114)	10.9% (11)

A total of 136 participants completed the pre‐survey and tutorial phase, with 124 completing fully and 12 submitting partial responses (Table 4). For Rounds 1–3, participant counts ranged from 124 to 128 completions, with small numbers of incomplete submissions in Rounds 1 (*n* = 3) and 3 (*n* = 4). In the post‐survey, 119 participants completed the full questionnaire, and 9 provided partial responses.

**Table 4:** Enrolment and completion.

	Pre‐survey and tutorial	Round 1	Round 2	Round 3	Post‐survey
Complete	124	125	128	124	119
Partially complete	12	0	0	0	9
Incomplete	0	3	0	4	0

Only eight participants who completed the tutorial session were unable to join a full CHAT session. These were all due to scheduling. Five of the participants did not show up for their tutorial and did not respond when we tried to reschedule, and three of the participants were unable to find a CHAT session during a time that was convenient for them (Table 4).

Discussions were generally equitable, as calculated using the HHI (Table 5).

**Table 5:** Enrolment and completion.

Number of deliberators in group	Range of participation, %*	Normalised HHI
5	16–23	0.00375
7	9–23	0.0232
4	23–26	0.0012
6	1–36	0.089
6	7–27	0.033
4	20–28	0.0047
3	17–41	0.059
5	16–28	0.012
5	12–24	0.012
5	13–31	0.028
5	11–41	0.075
4	18–29	0.010
5	9–36	0.052
4	16–32	0.023
5	8–38	0.081
6	11–23	0.013
8	4–21	0.0365
4	16–32	0.024
5	9–27	0.028
3	29–37	0.0054
3	32–35	0.0006
3	31–36	0.0015

* Proportions represent deliberators' participation in the discussion, calculated as #contributions to the discussion/all contributions. For example, if Deliberator JS made 10 contributions to a discussion that included 100 total contributions, JS would have contributed 10% of the deliberation.

### 3.1 Time

We aimed to keep sessions 3 hours long or less. In that amount of time, participants were able to consistently complete all three rounds of the CHAT exercise as well as their post‐survey.

### 3.2 Virtual Experience

When asked what they thought about the experience of discussing these topics and making these decisions as a group virtually versus face‐to‐face, participants provided both positive reasons for holding virtual sessions as well as drawbacks that they noticed.

### 3.3 Positives

#### 3.3.1 Comfort

Several participants stated that the virtual format allowed for more comfort. Those who tended to be introverted were emboldened to voice their opinions virtually but would not have if participating in person.

I'd say it makes it a little bit easier, at least for me, to speak up and share an opinion rather than being face‐to‐face with people.

So, for me, I tend to be pretty introverted, and I'm not sure I would've shown up quite the same way if we were all in person. There's something about being in the safety of my own home, having my own little mug of tea sitting here, and knowing I could just click my camera off or, you know, pretend I had technical difficulties or something if I got overwhelmed or I didn't like it.

Yeah, it's not as intimidating, too, like being a big group face‐to‐face.

I'm just gonna say I have a lot of social anxiety. So, for me to actually be in a place doing this would probably be really difficult for me, but being able to either turn on my camera or turn it off made me feel a lot more comfortable in being able to do this.

#### 3.3.2 New Paradigm

Some participants expressed that the pandemic has caused a paradigm shift, resulting in virtual sessions being perceived as the new standard. Therefore, hosting these sessions as virtual experiences was accepted as well as anticipated.

I have a new norm now. I've been so used to using Zoom and video, other, other video sources of video conference in our last couple or 3 years.

Like at the beginning of the pandemic, I did some virtual meetings, but not anywhere near where it is now. So, you know, you kind of come to expect things, you know.

I think that, at this point, a majority of people are kind of used to interacting over a virtual environment. So, I think that this website actually facilitated that pretty well.

It went pretty smoothly, and I think maybe most of us got so much experience in online meetings like this or stuff during COVID. Maybe if this was before that, I would've been a lot more confused and I wouldn't know what to do or how to work it, but I think the COVID year gave me enough experience and made me feel a little more comfortable with doing meetings this way now.

#### 3.3.3 Convenience

Participants also voiced that the virtual format provided added convenience. The virtual format eliminated commute time and allowed for more flexible scheduling.

I think also it's a little bit more convenient to work over the internet as well, just because, you know, when you're getting up you gotta get up, get dressed, go through traffic, find a parking space and all of that, and you just cut the middle man out, by being able to just come and sit in front of your laptop and do this. So…

The convenience was so much easier than me having to go somewhere else after a long day of work and then sit and do this. This I could walk around, do whatever I needed to do, and the convenience. The convenience is the biggest thing.

I think the convenience of being able to do it from my home, that I can schedule it around my day and I don't have to drive out to wherever a location it is since I kind of live in the middle of nowhere.

Right now, my car, it's in repair now. So, if ask me to go in person, that's probably not going to happen right now. Also, we can also invite a lot of people in Michigan.

My preference is virtual just because I have my kids with me, and so it makes it very difficult to find childcare at a certain date and time that might not work for a childcare provider.

### 3.4 Drawbacks

#### 3.4.1 Lack of Body Language

Some participants noted that one drawback to the virtual format was a lack of body language over the computer screen.

I mean, you usually get a lot more cues when it's face to face cuz you also have hand gestures and all that kind of stuff and body postures and we're just really looking from the shoulders up, but it's adequate.

you know, like the visual cues that you are missing because you're not in the same room together. It's not even the face. It's also like just picking up on things.

#### 3.4.2 Technology Problems

Technology problems were named as a barrier to participating in fully virtual sessions.

I will have one point that I think goes against that is tech problems. I might be biased in saying that I had tech problems. I think in person that would've…. I don't think I would've had tech problems in that way, but, you know, you win some, you lose some.

Personally, I had some issues with the screen, I had to exit and re‐enter the meeting, but other than that, I don't think we would've reached a different consensus when … if we would've met in person.

## 4 Discussion

In this paper, we evaluated the efficacy of a fully virtual public deliberation on the topic of priority setting after the Covid‐19 pandemic. We engaged individuals from underserved communities consistent with our aims since they are at risk for health disparities related to the social determinants of health and also likely to encounter problems related to the usability and accessibility of new software.

Overall, the results reveal substantial comfort with online deliberation. Those with social anxiety found it easier to participate and express themselves in studies conducted in online formats compared to face‐to‐face interactions. Furthermore, participants felt more encouraged to express their opinions with the group having the added shield of the virtual environment. These findings align with existing literature indicating that individuals with social anxiety often find online environments more conducive to participation than face‐to‐face settings [51]. One explanation could be due to online formats offering the benefits of reducing interpersonal pressure that participants often feel during in‐person activities, as found in a study by Mikhaylovskaya [52].

Convenience was also enhanced as the online format makes it easier for those with transportation barriers to participate. This finding is aligned with a study on online democratic participation during Covid‐19, which showed that offering digital formats significantly broadened public access [53]. Traditional focus groups tend to have participants of similar geographic demographics since the most convenience is provided to those who live close to where the focus group is held. The ability to participate from any location allowed for increased geographic diversity and allows participation of those who may otherwise struggle to access a physical venue, such as those with mobility issues [54, 55]. Additionally, groups remained within the estimated 3‐h time frame, and participants did not have to account for travel time, further aiding in increased convenience.

Participants also stated that there has been a shift towards a new paradigm where virtual spaces are much more common than in‐person ones. This is important to note because participants may expect a virtual option for participating in research going forward. Perhaps offering both in‐person and virtual options is the best approach to ensure participant preferences are being met. These findings are also supported by two‐thirds of participants' survey results stating that, if given the option, they would choose a virtual experience over a face‐to‐face one. Not only was the virtual format adequate, but it was also preferred by two‐thirds of participants in our study.

Many participants indicated that the 30‐min tutorial session before the full research session was helpful. This could indicate that virtual sessions are improved when participants familiarise themselves with the software before joining the deliberation. This is consistent with recommendations laid out by Lobe et al., who recommend completing one‐on‐one sessions with participants before the study [56]. Similarly, Laouris et al. found that virtual Structured Democratic Dialogue (SDD) could maintain or even enhance deliberative quality when sessions were well‐facilitated and participants were familiarised with the technology beforehand [9].

The technical limitations entailed in the process of this online deliberation did not seem to undermine the participatory experience. About half of the participants stated that no aspects of the video call technology could have been improved. The average rating towards the views of the exercise scale and the quality of deliberation scale were 3.3 and 3.5 on a 5‐point Likert scale (where 0 = Strongly Disagree and 4 = Strongly Agree), respectively. This indicates a generally positive sentiment of the virtual deliberation experience. The highest scored individual items were ‘I had lots of chances to share my views’, ‘during the exercise, I was treated with respect’, and ‘all positions were considered with equal respect’. This indicated that in the virtual environment, participants were able to voice their opinion in a respectful way, something that is important for public deliberations. This is consistent with current literature showing that well‐structured online deliberations can foster mutual respect, inclusive dialogue and collective learning [57, 58, 59]. High ratings for items like ‘I had lots of chances to share my views’ and ‘All positions were considered with equal respect’ reinforce the idea that the virtual CHAT format was able to provide a space for high‐quality deliberation. Compared to a prior version of the CHAT exercise that was completed face‐to‐face, participants rated their views of deliberation slightly higher in the virtual exercise [44]. Although this is not a direct comparison due to differences in the content of the exercise, this gives us insight into how the two modes of deliberation might compare. Overall, these results suggest no significant difference between in‐person and virtual formats in terms of the deliberative experiences of participants. Equality of participation, as calculated by the HHI, was evenly distributed amongst groups. Completion rates were high, with 82.6% of participants completing all study activities and 88.9% partially completing all study activities. Overall, these results suggest no significant difference between in‐person and virtual formats in terms of the deliberative experiences of participants.

## 5 Limitations

There were limitations based on who was able to participate. Some groups could be left out such as the older population due to limited internet literacy as well as remote persons due to struggles in accessing broadband internet [60]. The website on which CHAT was used is only compatible with laptops, Chromebooks or desktops. Although we tried to help volunteers find places (e.g., library and school), those with access to only smartphones or tablets were unable to participate. It is hard to determine how many participants this left out because this was a recruitment requirement. However, we did have participants find ways to gain access to these devices, such as utilising public libraries and borrowing devices from friends and family members.

We had a smaller number of Spanish‐speaking participants than we had anticipated. Despite using the same recruitment tactics as the English‐speaking sessions and expanding eligibility criteria to include individuals living outside of the state of Michigan, we still received very few screening surveys. One possible explanation is that Hispanic Americans are disproportionately affected by the digital divide (particularly regarding access to larger devices) and therefore may not have had the resources to access our recruitment materials in the first place [61]. Because our recruitment materials clearly stated the exercise would be fully virtual, some participants who knew they would have access issues may not have shown interest at all. Therefore, it is impossible to estimate how many people may have been left out for this reason. Including participants from outside the state of Michigan for the Spanish‐speaking groups introduced some confounding factors related to geographic and cultural variation, differences in local policy environments, and potentially differing access to resources and services. These contextual disparities were not explicitly controlled for in the analysis, which does not allow for the comparability of the results across groups.

This fully virtual version of CHAT, in its first project, had a few technical problems along the way. At times, these problems were disruptive to sessions; however, as the sessions went on, the software developers were able to find solutions to the problems and create a more stable website. Overall, the majority of participants reported CHAT to be a positive experience, and they were able to work through any technical issues that arose. The areas that participants singled out as warranting improvement were video quality, seeing and hearing presentations, and lagging and Wi‐Fi connection issues. Addressing these issues is a bit difficult since they have to do with individual device and internet situations. One possible solution is to recommend that participants utilise public spaces such as libraries for more reliable Wi‐Fi or that they borrow higher‐performing devices. These concerns are not uncommon in digital engagement and are particularly relevant when working with underserved populations, who may lack stable internet access or digital confidence [26, 62].

## 6 Conclusion

Online deliberations by low‐income adults about government spending priorities were feasible and rated highly. Our findings confirm that online formats can sustain meaningful engagement, openness to differing viewpoints, and respectful discourse. Participants identified increased comfort and convenience as key advantages of virtual deliberation, highlighting that individuals who face participation barriers may find online environments more accessible. The CHAT tool's design (three structured rounds with both individual and group decisions) translated effectively to the virtual setting and was well received by participants.

Additionally, the virtual mode expands opportunities for diverse and heterogeneous group compositions, promoting greater equity in voice and representation. This is likely due to a decrease in logistical barriers such as transportation and time constraints, which aligns with findings consistent in the current literature. It also reaches participants who are more willing to participate who otherwise would skip out had the session been held in‐person.

The virtual mode of these deliberations was identified as the new paradigm, generating more comfort and convenience than the traditional in‐person deliberations. Given the increasing normalisation of virtual communication accelerated by the Covid‐19 pandemic, this study underscores the importance of offering flexible participation modes. Allowing individuals to choose between in‐person and virtual deliberation could maximise inclusion and accommodate varying preferences and technological capabilities.

However, challenges remain, particularly regarding the lack of nonverbal communication cues and technological disruptions, which can affect the depth and flow of discussions. Addressing these technical and social nuances, and empirically comparing in‐person to virtual deliberations, is essential. Virtual modes of engagement could enable greater group heterogeneity and the participation of less mobile and more remote persons. Future studies could benefit by giving participants the option of participating in person or virtually so that they can choose what works best for their individual needs.

Finally, this study contributes to a broader conversation about how deliberative practices can adapt to new technological and social realities. As researchers and practitioners continue to experiment with formats ranging from synchronous video calls to AI‐enhanced moderation, our findings affirm that low‐tech, human‐facilitated virtual spaces remain valuable tools for meaningful democratic participation. This aligns with broader research on the ‘digital divide’, ensuring the most equitable access to participating in virtual research as possible.

In sum, virtual deliberations represent a promising evolution in participatory democracy, particularly for marginalised communities traditionally underserved by in‐person processes. Future research should continue to refine virtual tools and explore hybrid models that blend the strengths of both face‐to‐face and online engagement to build more accessible, equitable and effective deliberative platforms.

## Author Contributions

**Lydia Perry:** writing – original draft, data curation, project administration, writing – review and editing, formal analysis. **Gloria Carmona:** data curation, writing – original draft, writing – review and editing. **Marion Danis:** supervision, conceptualisation, investigation, funding acquisition, writing – original draft, writing – review and editing, methodology, validation, visualisation, formal analysis, project administration, data curation, resources. **Charo Ledón:** writing – review and editing, writing – original draft, data curation. **Maria Alvarez:** writing – original draft, writing – review and editing, data curation, formal analysis. **Zachary Rowe:** writing – original draft, writing – review and editing, data curation, formal analysis. **Susan Dorr Goold:** writing – original draft, writing – review and editing, funding acquisition, conceptualisation, investigation, methodology, formal analysis, data curation, supervision, resources, visualisation, validation, project administration.

## Conflicts of Interest

All authors have completed the ICMJE Form for Disclosure of Potential Conflicts of Interest. Dr. Susan Goold received consulting fees from the University of Colorado for serving as a Delphi panelist on comparing methods of community engagement. The same author also received honoraria for a talk at the McMaster International Review Course in Internal Medicine (MIRCIM) and participated in grant review activities for the Norwegian Centres of Excellence. Additionally, this author is involved in evaluations of the Michigan Medicaid expansion waiver for CMS/MDHHS, serves as a co‐investigator on a National Science Foundation planning grant, and holds NIH subcontracts on two projects: one with the University of North Carolina focused on Medicaid sterilisation policy and one with the University of Pennsylvania. None of these activities is related to the topic of this paper. The authors declare no other conflicts of interest.

## Data Availability

The data that support the findings of this study are available from the corresponding author upon reasonable request.
